# Identification of Novel Selective Transient Receptor Potential Vanilloid 4 (TRPV4) Agonists

**DOI:** 10.2174/0113816128381222250428094819

**Published:** 2025-05-02

**Authors:** Yuxin Jia, Meiying Liu, Heng Xu, Yixin Zhang

**Affiliations:** 1 Department of Burn and Plastic Surgery, Beijing Children's Hospital, Capital Medical University, Beijing, 100045, China;; 2 Department of Plastic and Reconstructive Surgery, Shanghai Ninth People's Hospital, Shanghai Jiao Tong University School of Medicine, Shanghai, 200011, China;; 3 Center of Medicinal Chemistry, Shanghai Institute of Materia Medica, Chinese Academy of Sciences, Shanghai, 201203, China

**Keywords:** TRPV4, GSK101, LM0038, mechanical pain, acute itch, sensory response

## Abstract

**Aims:**

We aimed to synthesize small-molecule compounds by modifying the chemical structure of GSK101 and screening for novel TRPV4 agonists with high specificity and selective sensory response.

**Background:**

GSK1016790A (GSK101) effectively activates Transient Receptor Potential Vanilloid 4 (TRPV4) and simultaneously induces mechanical allodynia and acute itch. However, as a commonly used tool compound for studying sensory function, its dual effects of pain and itch can interfere with each other.

**Objective:**

To design and synthesize a series of small-molecule compounds targeting TRPV4, evaluate their properties to identify the most specific tool compounds targeting TRPV4, and determine the correlation between TRPV4 activation and sensory response.

**Methods:**

In this study, live-cell Ca^2+^ imaging in a heterogeneous expression system was employed to evaluate the activity of synthetic compounds, molecular docking was performed to predict binding interactions and behavioral tests for itch and pain were combined with pharmacological and genetic strategies to assess physiological responses.

**Results:**

We synthesized nine GSK101 analogues and identified six small-molecule agonists that exhibited TRPV4-targeting excitability, preserved TRPV4-mediated mechanical pain perception, and attenuated the acute itch response.

**Conclusion:**

Our study provides new insight into the role of TRPV4 in pain and itch sensation and introduces LM0038, the most potent agonist, as a novel alternative to GSK101. With enhanced biological activity, it may serve as a valuable tool for studying TRPV4 function.

## 1 INTRODUCTION

Transient receptor potential vanilloid 4 (TRPV4) is a broadly expressed, polymodally gated, non-selective, calcium-permeable cation channel. It plays a critical role in various physiological processes, including osmolarity sensing [1], temperature regulation and maintenance [2, 3], bladder contractions [4], inflammation [5, 6], itch generation [7] and nociception [8]. These functions are implicated in multiple pathophysiological conditions, such as lung injury and asthma [9, 10], voiding dysfunction and bladder contractions [11], skeletal dysplasia [12], photosensitivity dermatitis and psoriasis [13], and neuropathic pain [14]. Therefore, developing TRPV4-targeting drugs has become a crucial strategy for studying relevant mechanisms and treating associated diseases.

TRPV4 channels can be directly activated by endogenous or exogenous chemical stimuli [15] and indirectly sensitized through intracellular signalling pathways [16]. Several synthetic pharmacological compounds targeting TRPV4 have been developed. The most well-known TRPV4 agonists include GSK1016790A (GSK101) [17] and the phorbol ester 4α-phorbol 12,13-didecanoate (4αPDD) [18]. Conversely, RN-1734 [19], HC-067047 [20], and GSK2193874 (GSK219) [21] are the most widely recognized TRPV4 antagonists. Notably, the highly potent TRPV4 channel blockers GSK2798745 have been evaluated in clinical trials involving patients with heart failure [22] and lung congestion [23]. In contrast, only the agonist GSK101 has been clinically tested to assess the effects of exogenous TRPV4 activation on human cutaneous vasodilatation and sweating. However, novel TRPV4 agonists remain in the research and development stage, and currently, no suitable agonists are available for clinical applications.

GSK101 is a piperazine-linked TRPV4 agonist that has been extensively used in *in vitro* cell and explant experiments to investigate physiological and pathological mechanisms, such as urinary bladder contraction [17], blood pressure regulation [24], suppression of proinflammatory signalling and cartilage degradation [25], and enhancement of myocardial ischemia-reperfusion injury [26]. As a result, it is widely employed in constructing animal models for TRPV4-related mechanistic studies. In terms of sensory perception, GSK101 activates TRPV4-mediated mechanical allodynia and acute itch [8]. This biological property makes GSK101 a key tool for studying TRPV4 in the sensory pathways of pain and itch. However, the relationship between the molecular structure of GSK101 and its activity remains unreported. Therefore, modulating TRPV4 activation by modifying the structure of GSK101 could represent a new pharmacological approach for TRPV4 agonist development.

With this in mind, we synthesized nine GSK101 derivates and identified a novel TRPV4 agonist, LM0038, as the most potent. Pharmacological studies showed that LM0038 specifically activated TRPV4-mediated calcium influx into different cell types. Compared to GSK101, LM0038 evoked a similar level of mechanical pain and less acute itch behaviours in mice. Given the improved biological properties of this new TRPV4 agonist, LM0038 may serve as a good candidate for the development of drugs targeting TRPV4 function.

## 2 MATERIALS AND METHODS

### 2.1 Reagents

GSK101, GSK219, ionomycin, dispase II, and bovine serum albumin (BSA) were purchased from Sigma-Aldrich (Burlington, MA, USA). The cDNAs for mouse TRPV4 were provided by the School of Pharmacy at South-Central Minzu University (Wuhan, China). The derived compounds were synthesized and provided by the Guofeng Xu Laboratory of the Shanghai Institute of Materia Medica, Chinese Academy of Sciences (Shanghai, China). GSK101, GSK219, and all synthetic compounds were dissolved to 30 mM with dimethyl sulfoxide (DMSO) and subsequently diluted with saline to the desired concentrations.

### 2.2 Animals

Eighty adult male wild-type C57BL/6J mice and thirty TRPV4 knockout C57BL/6J mice (weighing 23-25g) were purchased from Gem Pharmatech (Nanjing, China) and maintained under a 12-hour light-dark cycle at 24°C. For all experiments, mice aged 8 to 12 weeks were utilized and acclimatized to the experimental room one week before the experiments. The mice were assigned to the experimental and control groups (5 mice/group) using a computer-generated table of random numbers (0-10), as well as the sequence of dosing and measurement. All behavioral tests were recorded from a side view, and the evaluations were carried out by observers who were unaware of the animals' treatment and genotype.

### 2.3 HEK293 Cell Culture and Transfection

HEK293 cells were purchased from ATCC (ATCC CRL-1573; Manassas, VA, USA). As previously reported [27], the cells were tested for mycoplasma contamination before being cultured in the laboratory and grown as a monolayer in Dulbecco’s modified Eagle’s medium (DMEM) (Life Technologies, Carlsbad, CA, USA), supplemented with 10% fetal bovine serum (FBS; Life Technologies), 100 units/mL penicillin, and 100 μg/mL streptomycin. The cells were maintained in a humidified incubator at 37°C under 5% CO_2_. The cDNAs for mouse TRPV4 (mTRPV4) were transiently transfected into HEK293 cells for at least 24 hours using Lipofectamine 2000 (Invitrogen, Waltham, MA, USA).

### 2.4 Isolation and Culture of Murine Peritoneal Macrophages

Peritoneal macrophages were isolated as previously described [28]. The mice were euthanized by cervical dislocation, and their abdomens were dissected in the middle. Six milliliters of cold phosphate-buffered saline (PBS) was instilled into the peritoneal cavity, followed by gentle massage and subsequent aspiration. The samples were centrifuged for 5 min at 4°C and 300 g, resuspended in 2 mL PBS, and filtered using 70 μM cell strainers. After another centrifugation, the cell suspensions were seeded in 35 mM Petri dishes containing 8-9 culture slides and placed in a culture incubator (37°C, 5% CO_2_).

### 2.5 Isolation and Culture of Murine Primary Keratinocytes

Mice (P0-P2) were sacrificed and soaked in 10% povidone-iodine for 5 min. After being rinsed with 70% ethanol multiple times, the skin was removed and placed in a Petri dish containing PBS solution with 0.25% dispase II and incubated at 4°C overnight. The epidermis was then separated from the subcutaneous tissues, and keratinocytes were separated from the epidermis using a cell scraper and collected in 2 mL mouse keratinocyte media (Life Technologies). Dissociated cells were transferred to a 15% BSA column (1 mL) and centrifuged for 5 min at 1000 rpm. The cells were resuspended and seeded in 35 mM Petri dishes containing 8-9 culture slides and placed in the culture incubator.

### 2.6 Live-cell Ca^2+^ Imaging

Fura-2-based ratiometric measurements of intracellular calcium concentration ([Ca^2+^]_i_) were performed as previously described [27, 28]. HEK293T cells transiently transfected with mTRPV4, primary keratinocytes, and macrophages were loaded with 4 μM Fura-2 AM (Life Technologies) in culture medium at 37°C for 60 minutes. The cells were then washed three times and incubated in Hanks’ balanced salt solution (HBSS) at room temperature for 30 minutes before use. Fluorescence intensities were measured at excitation wavelengths of 340 nm and 380 nm using an inverted Leica fluorescence microscope equipped with excitation filter wheels (340 nm, 360 nm, and 380 nm). The data were captured using LAS X imaging software (Leica Microsystems, Wetzlar, Germany). The Fura-2 ratios (F340/F380) were used to indicate changes in intracellular calcium ion concentrations ([Ca^2+^]_i_) in response to stimulation. These values were derived from time-lapse imaging of each coverslip. The baseline of the cells was measured in the resting state, and once the value stabilized, the cells were treated with drugs. If no significant difference in the cell baseline was observed, cell viability was considered consistent. The activation threshold was defined as three standard deviations (SDs) above the mean, which corresponds to approximately 20% above the baseline.

### 2.7 Dose-response Assessments

GSK101 and the compounds were dissolved to create a concentration gradient (0.3 nM, 3 nM, 30 nM, 100 nM, 0.3 μM, 3 μM, 30 μM, 300 μM). Live-cell calcium imaging experiments were performed using fura-2-labeled TRPV4-HEK293 cells, and the fluorescence curves were recorded for each concentration of the compounds. The experiments were repeated three times, with the number of live cells in each field of view exceeding 100. The real-time fluorescence intensity curves from each experiment were statistically analyzed to calculate the cell response rate (the percentage of cells showing a fluorescence increase greater than 20% above baseline relative to the total number of cells). The concentration-response curves were fitted using GraphPad Prism (with the logarithm of concentration on the X-axis and the response rate on the Y-axis), and the Hill coefficient and EC_50_ values for each compound were automatically output.

### 2.8 Molecular Docking

In this study, two molecular docking tools were utilized to predict the binding modes of the small molecule compound LM0038 with the TRPV4 protein. Initially, preliminary docking was performed using AutoDock Vina software, with the grid center set to cover the active site of the TRPV4 protein, followed by conformational searching. Subsequently, high-precision docking was conducted using the Glide module (Schrödinger Suite) to optimize the binding conformation, analyze binding free energy and key interactions, and evaluate binding affinity through Glide Score and Glide Emodel.

### 2.9 Mechanical Pain Test

The mice were allowed to acclimate for 1.5 hours in red plastic enclosures on a metal mesh platform. The test compounds (30 μM) were formulated in 0.9% saline solution and administered *via* intraplantar injection with a volume of 10 μL. Calibrated von Frey filaments (0.02, 0.04, 0.07, 0.16, 0.4, 0.6, 1.0, 1.4, and 2.0 g) were applied perpendicularly to the plantar surfaces of the mice's hind paws for up to 2 seconds, with sufficient force to cause the filament to bend [27]. Brisk withdrawal or flinching of the hind paw during or immediately after application was considered a positive response. Mechanical hyperalgesia was assessed both before (baseline) and 3 hours after the injection of test compounds. The threshold force required to elicit paw withdrawal (50% mechanical threshold) was obtained using the up-down method [29, 30].

### 2.10 Acute Itch Test

Under 1 to 2% isoflurane anesthesia, the mice were shaved on the nape of the neck 5 to 7 days prior to the experiment. For all itch behavior experiments, the mice were acclimated in red plastic chambers for two days before measurements, with acclimation sessions lasting 2 hours on the first day and 1.5 hours on the day of measurement. GSK101 (30 μM) and the test compounds (30 μM and 300 μM) were formulated in 0.9% saline and administered *via* intradermal injection to the nape of the neck at a volume of 50 μL. Video recordings were conducted for 30 minutes after the injection in the absence of any observers. Acute chemical itch was evaluated by counting the number of scratches within the 30-minute recording period.

### 2.11 Statistical Analysis

All data are presented as mean ± standard error of the mean (SEM) for n independent observations. Data were excluded from statistical analyses only for technical errors. Student’s t-test was employed to assess the statistical significance between the two groups. ANOVA tests were utilized to evaluate hypotheses concerning effects across multiple groups. All tests were performed as 2- tailed tests. A *p*-value of less than 0.05 was considered to indicate a statistically significant difference. Prism 10 (GraphPad Software, Boston, MA, USA) was used for all statistical analyses.

## 3 RESULTS

### 3.1 Chemical Synthesis of GSK101 Derivatives and Evaluation of their Capacity to Activate TRPV4 in Cell-based Assays

**Fig. (1):**
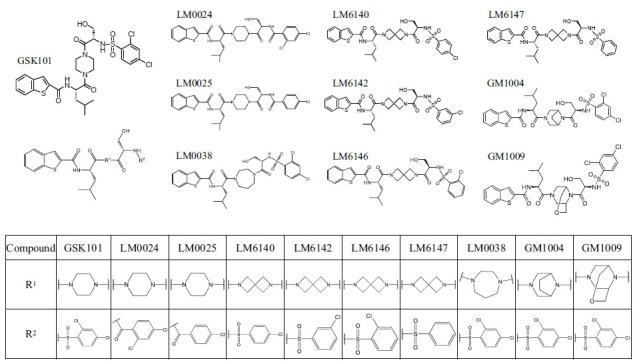
Modification of GSK101 for improved targeting of TRPV4. We synthesized nine small-molecule derivatives by modifying the chemical groups on both sides of GSK101, namely, the piperazine at the R^1^ position and the sulfonyl group at the R^2^ position. For compounds LM0024 and LM0025, the R^1^ group was modified to a carbonyl group, and the R^2^ group was respectively replaced by an ortho/para benzene ring and a para chlorine atom. For compounds LM6140, LM6142, LM6146 and LM6147, the R^1^ group was replaced by 2,6-diazaspiro[3.3]heptane, and a new benzene ring with a halogen atom in different positions from those of GSK101 was used instead of the original R^2^ group. For compounds GM1009 and GM1004, the R^2^ group remained unchanged, while macrocyclic and bridged ring groups took the place of the R^1^ group.

Based on the chemical structure of GSK101, we synthesized nine small-molecule compounds with subtle modifications (Fig. **1**). Inspired by GSK101; we started with individual chemical groups to avoid the possibility that large structural modifications changed the activity. We first retained the piperazine at the R1 position and modified the R2 group. We replaced the sulfonyl group of GSK101 with a carbonyl group and substituted it with an ortho/para benzene ring and a para chlorine atom to form the starting compounds LM0024 and LM0025. We then synthesized compounds LM6140, LM6142, LM6146, and LM6147 by replacing the R1 group with 2,6-diazaspiro[3.^3^]heptane to explore the effect of the halogen atom substituent positions on the benzene ring of the R2 group. In addition, keeping the structure of R2 unchanged, we modified the R1 group with macrocyclic or bridged ring groups to produce the compounds GM1009 and GM1004. All nine GSK101 derivatives were examined experimentally.

### 3.2 GSK101 Derivatives Activate Recombinant TRPV4 in Heterologous Cells

To evaluate the efficacy of these synthetic analogues *in vitro*, we assessed their ability to activate TRPV4 using a Ca^2+^ imaging assay. Because of the low expression of TRPV4 in native cells, we utilized a heterologous expression system and conducted Ca^2+^ imaging in HEK293T cells that were transiently transfected with mouse TRPV4 (TRPV4-HEK293 cells).

**Fig. (2):**
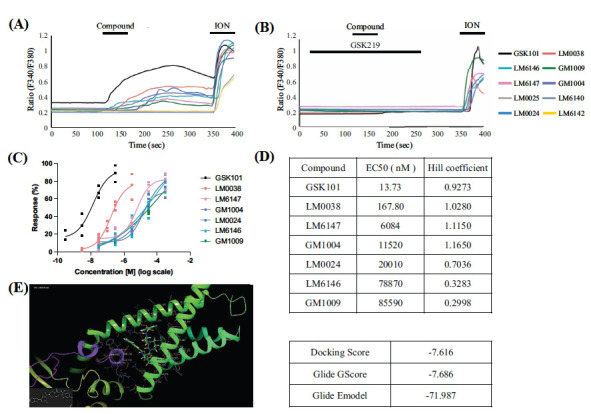
Ca^2+^ imaging of GSK101 and its synthetic derivatives in TRPV4-HEK293 cells and the molecular docking of LM0038 with TRPV4. (**A**) Flura-2 Ca^2+^ imaging of GSK101 and each derivative in TRPV4-HEK293 cells with directed expression of TRPV4 (mouse) after transfection. The cells were stimulated with TRPV4-selective activator compound, GSK101, and the 9 derivatives at a concentration of 30 nM for 2 min. After 2 minutes of cell flushing, the cells were stimulated by 1 μM ionomycin. The curve shows the average fluorescence ratio of cells on each slide at 340 and 380 nm over time, where the peak indicates an immediate calcium influx. Except when treated with LM0025, LM6140 and LM6142, the cells showed obvious calcium influx. The rapid response after the administration of ionomycin demonstrated the good viability of TRPV4-HEK293 cells. (**B**) Live-cell Ca^2+^ imaging of compounds was repeated in the presence of 100 nM GSK219. Preapplied and coapplied GSK219 abolished the action of GSK101 and compounds. (**C**) Dose-response of GSK101 and other TRPV4-activating compounds in TRPV4-HEK293 cells. (**D**) The EC_50_ values were 13.73 nM (GSK101), 167.80 nM (LM0038), 6084 nM (LM6147), 11520 nM (GM1004), 20010 nM (LM0024), 78870 nM (LM6146) and 85590 nM (GM1009). The Hill coefficients for the agonists are 0.9273 (GSK101), 1.0280 (LM0038), 1.1150 (LM6147), 1.1650 (GM1004), 0.7036 (LM0024), 0.3283 (LM6146) and 0.2998 (GM1009). (**E**) Molecular docking binding mode diagram of LM0038 with TRPV4 and Glide comprehensive scoring.

Firstly, the agonist GSK101 and each of the 9 synthetic compounds were used at 30 nM (Fig. **2A**). Two minutes after administration, LM0025, LM6140, and LM6142 produced no measurable current. GSK101 and the other six compounds produced a Ca^2+^ influx of different strengths, manifested as robust fluctuations in the ratio of 340 and 380 nm fluorescence signals. TRPV4-HEK293 cells were then treated with 1 μM ionomycin (ION) as a positive control, which rapidly and significantly increased intracellular calcium concentration, thereby verifying the effective operation of the experimental system (comprising cells, fluorescent probes, and imaging equipment).

To determine the specificity of the GSK101 derivatives, we used the selective antagonist compound GSK219. As expected, the calcium influx activated by GSK101 and the other six compounds was abolished in the presence of GSK219 (Fig. **2B**), suggesting that compounds LM0038, LM6147, GM1004, LM0024, LM6146, and GM1009 act as selective agonists capable of activating TRPV4-dependent calcium influx, thereby demonstrating TRPV4 targeting and activation in heterologous cells.

Subsequently, we performed more detailed dose-response assessments for compounds LM0038, LM6147, GM1004, LM0024, LM6146 and GM1009, which yielded EC_50_ values of 167.80 nM, 6084 nM, 11520 nM, 20010 nM, 78870 nM and 85590 nM, respectively. In contrast, the control agonist GSK101 exhibited an EC_50_ of 13.73 nM (Fig. **2C**). These findings indicated that most compounds could effectively activate TRPV4 channels and initiate calcium influx at optimal concentrations, with LM0038 being the most potent. Compared with GSK101, the drug activity of the modified compounds was reduced by at least tenfold, and three of the compounds even lost their activity. Notably, the chemical structure modifications did not compromise the TRPV4 targeting of GSK101.

The Hill coefficient value of the concentration-response curve for compound LM0038 is close to 1 (Fig. **2D**), indicating that its binding site on the TRPV4 molecule is relatively independent and does not involve complex multi-subunit cooperative effects. To validate this hypothesis, we conducted molecular docking experiments using AutoDock Vina and Glide to analyze the interaction between LM0038 and the TRPV4 molecule. The results showed that the ligand LM0038 tightly binds to the active site of the TRPV4 protein, forming hydrogen bonds with the carbonyl oxygen of ASN474 and the hydroxyl hydrogen of THR527 (Fig. **2E**). These residues constitute the key binding pocket responsible for the activation of TRPV4. The molecular docking scoring reveals that LM0038 demonstrates high binding affinity to TRPV4, with a highly stable binding conformation and energy state, as well as strong interactions. This suggests that LM0038 could serve as an effective TRPV4 agonist.

### 3.3 GSK101 Derivatives Activate TRPV4-expressing Primary Cells

Next, we compared the potency of the most potent compound, LM0038, with that of the parental compound, GSK101, in two types of primary cells known to express TRPV4, where TRPV4 function has been demonstrated in a relevant biological context. We examined neonatal mouse keratinocytes, which have prominent TRPV4 expression and whose TRPV4 channels participate in the release of neuroactive mediators and regulation of the inflammatory response. In addition, we studied murine peritoneal macrophages, in which TRPV4 modulates both inflammatory and anti-inflammatory functions through its role in phagocytosis, cytokine release, and regulation of signaling processes [5].

**Fig. (3):**
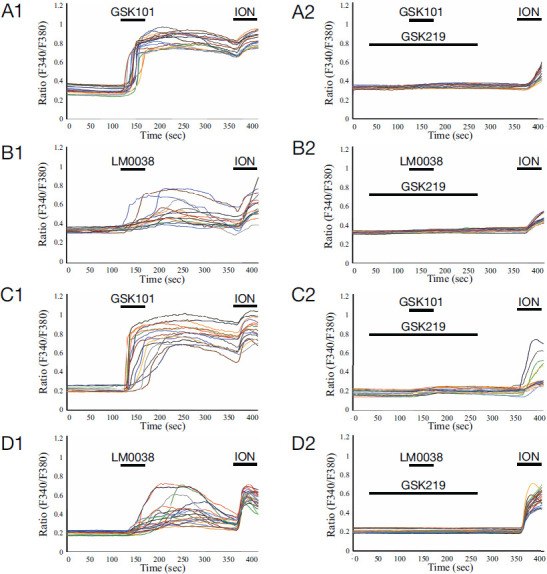
Ca^2+^ imaging of compounds in TRPV4-keratinocytes and macrophages. (**A**) Representative plots showing the GSK101 (0.3 μM)-elicited [Ca^2+^]_i_ responses in primary keratinocytes of neonatal TRPV4^+/+^ mice. Pre- and co-applied GSK219 (1 μM) abolished the GSK101 action. (**B**) GSK101 was replaced by LM0038 (0.3 μM). Repeated experiments in TRPV4^+/+^ primary keratinocytes showed weaker calcium influx than before, and the signal was abolished in the same way. (**C**) Representative plots showing GSK101 (0.3 μM)-elicited [Ca^2+^]_i_ responses in freshly dissociated peritoneal macrophages from TRPV4^+/+^ mice. Pre- and co-applied GSK219 (1 μM) abolished the GSK101 action. (**D**) GSK101 was replaced by LM0038 (0.3 μM). Repeated Ca^2+^ imaging of TRPV4^+/+^ primary peritoneal macrophages showed signals with lower peaks than before, and the signals were abolished in the same way.

In these two cell types, GSK101 and its derivative, LM0038, induced a significant TRPV4-mediated calcium response, which was inhibited by GSK219 (Fig. **3**). The peak traces of Ca^2+^ imaging and the ratio of activation suggested lower potency for LM0038 compared to GSK101.

These results suggested that compound LM0038 was a TRPV4-activating compound with micromolar potency in heterologous systems, as well as in TRPV4-expressing primary keratinocytes and macrophages, with lower potency than the parental molecule GSK101. These modifications of the chemical structure of GSK101 did not yield a positive effect on enhancing the potency of the agonist.

### 3.4 Six of the Derived Compounds Induced TRPV4-dependent Pain Hyperalgesia

As a selective TRPV4 agonist, GSK101 induces mechanical allodynia as well as intense acute chemical itch in mice. This dual sensitivity can complicate experiments aimed at studying pain or itch independently. Therefore, to determine whether the new compounds exhibit more precise pain- or itch-inducing effects, we evaluated their performance in animal behavioral models.

**Fig. (4):**
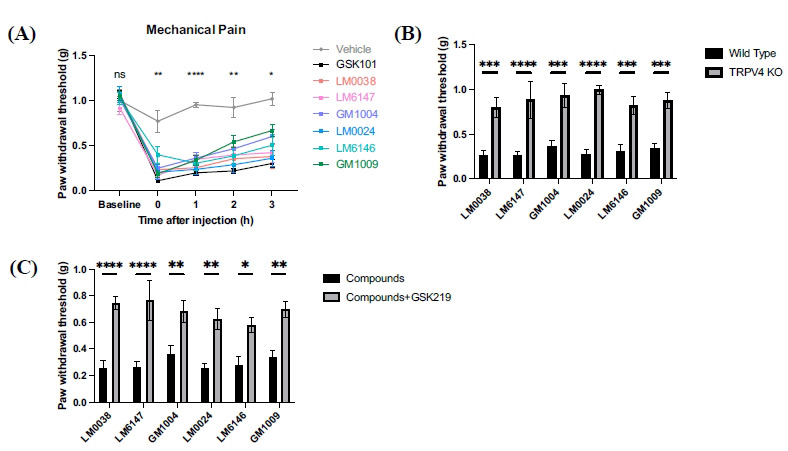
TRPV4-targeting derivatives effectively induced mechanical allodynia. (**A**) Intraplantar injection of GSK101 or the small-molecule agonist (30 µM, 10 µL per mouse) significantly induced mechanical hyperalgesia compared to the control group (n = 6). The immediate effect of LM6146 was notably weaker than that of GSK101 (*p =* 0.0413), but its pain-inducing effect reached a level comparable to GSK101 within one hour (*p =* 0.8668). (**B**) Intraplantar injection of agonists in TRPV4-knockout mice induced less paw-withdrawal behavior than that observed in wild-type mice (n = 4-6). (**C**) The TRPV4 inhibitor GSK219 was injected intraperitoneally 30 minutes before plantar injection of the synthetic compounds (30 µM, 100 µL/mouse). The mechanical pain threshold of each compound was significantly raised (n = 4-6). Data are presented as means ± SEM. *p < 0.05, **p < 0.01, ***p < 0.001, ****p < 0.0001, ns, not significant, ANOVA. (A. ^[0h]^*p =* 0.0062, ^[1h^^]^*p* < 0.0001, ^[2h^^]^*p* = 0.0048, ^[3h^^]^*p =* 0.0110. B. ^[LM6147, LM0024]^*p* < 0.0001, ^[LM0038]^*p =* 0.0003, ^[GM1004]^*p =* 0.0002, ^[LM6146]^*p =* 0.0007, ^[GM1009]^*p =* 0.0004. C. ^[LM0038, LM6147]^*p =* < 0.0001, ^[GM1004]^*p =* 0.0099, ^[LM0024]^*p =* 0.0019, ^[LM6146]^*p =* 0.0167, ^[GM1009]^*p =* 0.0031)

We first assessed these compounds in an *in vivo* model of irritant pain in wild-type mice. Injection of 30 µM TRPV4-targeted compounds into the plantar region of the hind paw elicited significant paw withdrawal responses compared to the control group, with pain sensations lasting for over 3 hours. Statistical analysis revealed that the immediate pain-inducing effect of LM6146 was slightly weaker than that of GSK101, but the strong pain-inducing effects of all compounds one-hour post-injection showed no significant difference compared to GSK101 (Fig. **4A**).

We repeated the above experiments using TRPV4-knockout mice. After subcutaneous injection of the compounds, the knockout mice displayed significantly less pain behavior compared to the wild-type mice (Fig. **4B**). Moreover, the TRPV4 inhibitor GSK219 blocked the pain-like behavior induced by GSK101 derivatives (Fig. **4C**). These results indicated that mechanical allodynia was significantly reduced with suppressed TRPV4 function, verifying the direct role of TRPV4 channels in mechanical pain.

### 3.5 Six of the Derived Compounds Induced Acute Itch with a Lower Degree than GSK101

**Fig. (5):**
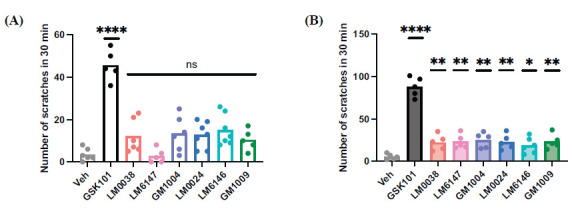
TRPV4-targeting derivatives induced acute itch in a dose-dependent manner. (**A**) Intradermal injection into the nape of either vehicle or small-molecule agonists (30 µM, 50 µL/mouse) induced fewer than 15 scratches on average. As positive control, GSK101 caused intense chemical itch with an average of 50 scratches (n = 5-8). (**B**) High concentration (300 µM, 50 µL/mouse) of agonists caused significantly increased acute itch. GSK101 caused an average of 88 scratches, while the other compounds averaged around 25 (n = 5). Data are presented as means ± SEM. *p < 0.05, **p < 0.01, ***p <0.001, ****p < 0.0001, ns, not significant, ANOVA. (A. *p*< 0.0001. B. ^[GSK101]^*p* < 0.0001, ^[LM0038]^*p =* 0.0068, ^[LM6147]^*p =* 0.0041, ^[GM1004]^*p =* 0.0011, ^[LM0024]^*p =* 0.0047, ^[LM6146]^*p =* 0.0398, ^[GM1009]^*p =* 0.0036).

TRPV4-targeting small molecules were then applied to a mouse model of acute itch. GSK101 and the derived compounds were administered *via* intradermal injection (30 μM) into the nape of wild-type mice, and a 30-minute video recording was obtained. After reviewing the videos, we quantified the number of scratching bouts induced by GSK101 and the six other compounds. The results showed that GSK101 elicited frequent scratching behaviors, with an average of 50 scratching bouts within 30 minutes. In contrast, the other six compounds at the same concentration caused fewer than 15 scratching bouts on average, a level comparable to that of the vehicle group (Fig. **5A**).

We further increased the concentration of the compounds to 300 μM and repeated the acute itch experiments. The results demonstrated that all six compounds induced more scratching, with an average of 20 bouts within 30 minutes, indicating a higher degree of acute itch (Fig. **5B**). This suggested that increasing the concentration of the compounds effectively enhanced their itch-inducing effects. However, after the concentration increase, the itch response of GSK101 was also intensified. In other words, the acute itch response induced by the derived compounds was consistently lower than that of GSK101.

Overall, all six selective agonists retained the TRPV4-related pain-inducing properties of GSK101. Simultaneously, the structural modifications of these synthetic small molecules attenuated the itching potency of GSK101, eliminating the dual effect of pain and itch. This singular sensitivity enables these compounds to be more precisely applied in animal models and to function more accurately in TRPV4-related studies.

## 4 DISCUSSION

In this study, we employed a heterogeneous expression system and various pharmacological synthetic compounds for cellular-level investigations. We identified six small molecules (LM0038, LM6147, GM1004, LM0024, LM6146, and GM1009) that successfully evoked a TRPV4-dependent [Ca^2+^]i response in HEK293T cells transiently transfected with mTRPV4. These findings demonstrate the deasibility of chemical synthesis and screening based on the structural formula derived from the chemical structure of GSK101. Furthermore, to enhance the completeness and reliability of the *in vitro* results, we evaluated the TRPV4-activating ability of GSK101 and the most potent derived compound, LM0038, using two different types of murine primary cells: keratinocytes and macrophages.

In ethological testing, the six molecules induced analogous mechanical allodynia in a TRPV4-dependent manner and triggered acute itch in a dose-dependent manner. These small molecules mediated pain responses at an intensity comparable to GSK101 but significantly attenuated GSK101-mediated pruritus, thereby simplifying the dual sensory effects of GSK101. Among the six synthetic compounds, we identified LM0038 as the most promising small- molecule analogues of GSK101.

Keratinocytes reside near the peripheral terminals of nociceptors and produce various neuroactive mediators, such as ATP, interleukin-1β, prostaglandin E2, endothelin, and NGF, which are known to elicit pain [31]. During inflammation and injury, TRPV4 channels expressed in keratinocytes are activated, leading to the production of inflammatory mediators such as IL-6, IL-8 and endothelin, as well as various neuropeptides including substance P and (CGRP) [32-35]. Simultaneously, the activation of TRPV4 in macrophages induces calcium influx, promoting the release of inflammatory factors such as TNF and IL-1β [31, 36], and mediating macrophage phagocytosis, the secretion of pro-resolution cytokines, and the generation of reactive oxygen species [5]. The cytokines secreted by both cell types act on sensory neurons in the adjacent skin, triggering neural pathological pain circuits, transmitting signals to the brain and eliciting the sensation of pain [8, 37, 38]. These cytokines are also involved in macrophage-mediated immune regulation [6, 39], forming neuro-immune interactions that enhance cytokine release and sensitize nerve fibers, thereby synchronously participating in the transmission of pain signals [40, 41].

**Fig. (6):**
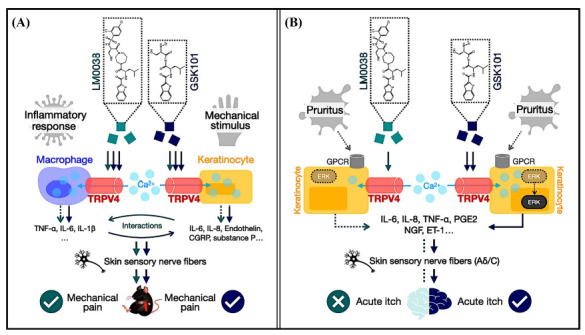
Schematic diagram of the mechanisms of pain and itch responses mediated by LM0038 and GSK101. (**A**) GSK101 and LM0038 activate TRPV4 channels, leading to calcium influx-mediated neuro-immune interactions and triggering pain responses. (**B**) GSK101 and LM0038 activate the TRPV4 channels in keratinocytes, inducing varying degrees of calcium influx-mediated cytokine release. The strong agonist GSK101 triggers an itch response, while the TRPV4 activation by LM0038 is insufficient to induce an itch response.

Based on the functional responses of murine keratinocytes and macrophages to GSK101 and LM0038, as well as their pain-inducing effects in animals, it is reasonable to speculate that TRPV4 acts as both a pain transducer and an inflammatory regulator. TRPV4 mediates not only the perception of noxious stimuli through keratinocytes but also the inflammatory response induced by macrophages. In other words, TRPV4 agonists can activate TRPV4 channels and directly induce local pain sensations *via* neuroimmune cooperation (Fig. **6A**). Although less potent than GSK101, the synthetic compound can elicit pain responses comparable to GSK101 as long as TRPV4 is effectively activated. It is conceivable that agonists more potent than GSK101 may induce acute pain at a similar level, or alternatively, result in pain paralysis due to excessive TRPV4 activation.

The TRPV4-mediated interactions between the nervous and immune systems, as well as the role of TRPV4 in other cell types potentially involved in pain sensation, remain poorly understood. It is important to note that TRPV4 participates in multifactorial pain perception, including inflammatory pain [6, 42] and mechanically evoked pain [8]. Various animal models, such as temporomandibular disorder and skin inflammation pain models, could be utilized for future compound screening and may further validate the pain-inducing properties of TRPV4 agonists.

Itching is a more complex sensation than pain. Exogenous pruritogens, such as chloroquine, and endogenous pruritic mediators, such as histamine and serotonin (5-HT), are sensed by G protein- coupled receptors (GPCRs), which then modulate downstream effectors, including ion channels and enzymes [43]. In epidermal keratinocytes, TRPV4 functions as a pruriceptor channel downstream of GPCR in acute itch, while direct activation of TRPV4 channels also induces scratching behavior, which appears to be entirely dependent on TRPV4 expression [8]. Calcium influx through TRPV4 upregulates the phosphorylation of extracellular regulated protein kinases (ERK) in keratinocytes, which are then converted into sensory signals and generate itch of varying intensity. As observed in primary murine keratinocytes, the compound LM0038 and GSK101 activated TRPV4-associated calcium influx to different extents and intensities, and this difference in potency explains their distinct itch responses (Fig. **6B**). However, all primary cells in our study were of mouse origin, which limits the translational understanding and application of these findings to human TRPV4.

Nociceptive and innocuous mechanical signals are processed by the somatosensory system of the brain, resulting in two fundamentally distinct sensations - pain and itch - that elicit different behavioral responses, such as withdrawal and scratching [44]. Clinically, itch is often accompanied by pain, and a certain degree of pain can trigger scratching behavior similar to that caused by itch. However, itch dose not always coexist with pain. In some diseases, such as shingles, pain can exist independently. Therefore, pain and itch are two distinct yet closely related sensations. Studying pain and pruritus separately can help avoid confusion arising from similar behavioral manifestations of different sensations. It is essential to optimize the dual role of GSK101 in the development of agonists and the screening of tool compounds. The specific sensory response of LM0038 represents a significant advantage in drug synthesis highlighted in this study.

Additionally, TRPV4 cooperates with other channels, such as transient receptor potential A1 (TRPA1), to participate in signal transduction and sensory perception. For example, the co-involvement of TRPV4 and TRPA1 plays a role in several health conditions, including colitis, itch, respiratory injuries, and chronic cough [45]. Evaluating the specificity of these compounds against a broader range of receptors and ion channels will be the focus of future studies. Furthermore, investigating how these TRPV4 agonists are administered and interact with different channels represents a promising translational direction for research.

## CONCLUSION

As a widely used pharmacological tool, GSK101 activates the TRPV4 channel and induces acute pain and itch sensations in mice. However, GSK101 fails to differentiate between itch and pain, which limits its application. To address this limitation, in this study, we synthesized a series of small-molecule compounds by modifying the chemical structure of GSK101 and screened for novel TRPV4 agonists with high specificity and selective sensory responses. Through calcium imaging, molecular docking and behavioral tests, we identified the newly synthesized compound LM0038 as a TRPV4-targeting agonist with excitability in various cell types. LM0038 not only preserves TRPV4-mediated mechanical pain perception but also attenuates the acute itch response. We propose that LM0038 could serve as a novel alternative to GSK101, reducing the overlap between pain and itch for studying TRPV4 function.

## Data Availability

The data and supportive information are available within the article.

## LIST OF ABBREVIATIONS

BSA: Bovine Serum Albumin
DMSO: Dimethyl Sulfoxide
GPCRs: G Protein-coupled Receptors
ION: Ionomycin
TRPA1: Transient Receptor Potential A1
TRPV4: Transient Receptor Potential Vanilloid 4
